# On the physiology of jouissance: interpreting the mesolimbic dopaminergic reward functions from a psychoanalytic perspective

**DOI:** 10.3389/fnhum.2013.00709

**Published:** 2013-11-06

**Authors:** Ariane Bazan, Sandrine Detandt

**Affiliations:** ^1^Service de Psychologie Clinique et Différentielle, Université Libre de Bruxelles (ULB), BruxellesBelgium; ^2^Stellenbosch Institute for Advanced Study, Wallenberg Research Centre at Stellenbosch UniversityStellenbosch, South Africa; ^3^Research fellow Fonds de la Recherche Scientifique FNRS-FRESH, Service de Psychologie Clinique et Différentielle, Université Libre de Bruxelles (ULB),Belgium

**Keywords:** neuropsychoanalysis, jouissance, enjoyment, Lacan, addiction, reward, dopamine, psychoanalysis

## Abstract

*Jouissance* is a Lacanian concept, infamous for being impervious to understanding and which expresses the paradoxical satisfaction that a subject may derive from his symptom. On the basis of Freud’s “experience of satisfaction” we have proposed a first working definition of *jouissance* as the (benefit gained from) the motor tension underlying the action which was [once] adequate in bringing relief to the drive and, on the basis of their striking reciprocal resonances, we have proposed that central dopaminergic systems could embody the physiological architecture of Freud’s concept of the drive. We have then distinguished two constitutive axes to *jouissance*: one concerns the subject’s body and the other the subject’s history. Four distinctive aspects of these axes are discussed both from a metapsychological and from a neuroscience point of view. We conclude that *jouissance* could be described as an accumulation of body tension, fuelling for action, but continuously balancing between reward and anxiety, and both marking the physiology of the body with the history of its commemoration and arising from this inscription as a constant push to act and to repeat. Moreover, it seems that the mesolimbic accumbens dopaminergic pathway is a reasonable candidate for its underlying physiological architecture.

## INTRODUCTION

We previously proposed physiological frameworks to understand a number of psychoanalytic concepts like repression and primary process, as well as the Lacanian concept of the signifier (Bazan, 2006, 2007, 2009, 2011, 2012; Bazan and Snodgrass, 2012). The topic of the present paper will be another Lacanian concept, *jouissance*, which is quite untranslatable but has been translated before as *enjoyment* (Evans, 1996). The concept itself is also infamous for being impervious to understanding. Because the concept appears relatively late in the teaching of Lacan, and only by bits and pieces, we chose to start with a clinical description. Indeed, clinical experience leads us to unmistakably identify a human tendency to seek, beyond the mere pursuit of pleasure, for that which brings the subject into danger or for that which sabotages his life. In its purest clinical form *jouissance* is what explains why people are addicted to harmful, or even lethal, substances – e.g., why people cannot stop smoking even after being diagnosed with lung cancer. *Jouissance* thus expresses the paradoxical satisfaction that the subject derives from his symptom (Evans, 1996, p. 92). In *Mourning and melancholia*, for example, Freud (1917/1914–1916, p. 251) says literally: “The self–tormenting in melancholia, (which) is without doubt enjoyable:” the melancholic subject also gains satisfaction from self–devaluation. *Jouissance* is a crucial concept for clinics, as it explains why, against all rationality, subjects are often wedded to their problems, be it at the highest price, i.e., at the cost of their professional career, of their relationships or of their mere lives.

In the early seminars, Lacan (1975/1953–1954; 1978/1954–1955) uses the term with a reference to its original, juridical, meaning: the term arose in the XV century, to designate *the action of using a property for the purpose of obtaining the satisfaction it is supposed to provide*. It is akin in its meaning to the juridical concept of “usufruct,” which is a *right of enjoyment, enabling a holder to derive profit or benefit from property that either is titled to another person or which is held in common ownership, as long as the property is not damaged or destroyed*. There is an essential distinction to take from these juridical definitions, which founds the notion of *jouissance*: it is the distinction between the satisfaction of *consuming* something, whereby it could be damaged, destroyed or lost in the consumption, and the satisfaction of *using* something with this satisfaction being explicitly not tied to its consumption. Several Lacanian authors explicitly use this definition of *jouissance* in its original juridical reference: e.g., Robin (2006, p. 29), in the context of addiction, defines enjoyment as the profit one can obtain from something which he does not possess. Jadin (2012/2009, p. 42) explains that abuse could be defined as “treating the body of the child as if one had usufruct of it.”

Later on in Lacan’s work, the sexual connotations of *jouissance *become more apparent. It is in *The Ethics of Psychoanalysis*, then, that (Lacan, 1986/1959–1960, p. 209) proposes that “jouissance appears not purely and simply as the satisfaction of a need, but as the satisfaction of a drive.” Indeed, up to 1957, the term seems to mean no more than the enjoyable sensation that accompanies the satisfaction of a biological need such as hunger (Lacan, 1994/1956–1957, p. 125), but in this seminar *jouissance* and pleasure are distinguished. It is therefore that, starting precisely from Freud’s model of drive, we will propose a metapsychological understanding of the concept of *jouissance* which will allow for an operationalisation in physiological terms. We have organized our paper in two parts, a psychoanalytic (metapsychological) part followed by a (neuro–)physiological part. Moreover, we have distinguished two constitutive axes to *jouissance*: one has to do with the *body* and entails the aspects of (1) the drive, (2) the experience of satisfaction and (3) the dimension of excess (of body tension); the other has to do with the (subject’s) *history* and basically entails the commemoration of a trait, complying to repeat. These four distinctive aspects are respectively discussed both from a metapsychological and from a neuroscience point of view.

## METAPSYCHOLOGY: FROM BODY TO HISTORY

### BODY

#### Freud’s model of the drive

In Freud’s (1915) model, a drive has a source (a biological need or lack), an aim (the satisfaction of that need), an object (adequate in satisfying the need) and an impetus (a pressure pushing to act). Hunger could, in this model, start with a biological signal, such as low blood sugar sensed in the lateral hypothalamus (LH). This lack is sensed by the central nervous system (symbolized by ψ in Freud’s *Project*) where the lack accumulates as an excess of endogenous quantities: “The nucleus of ψ is connected with the paths by which endogenous quantities of excitation ascend. (…) The filling of the nuclear neurones in ψ will have as its result an effort to discharge, an *urgency* which is released along the motor pathway. Experience shows that here the first path to be taken is that leading to *internal change* (expression of emotions, screaming, vascular innervation). But no such discharge can produce an unburdening result, since the endogenous stimulus continues to be received and the ψ tension is restored” (Freud, 1956/1895a, p. 317–318). In other words, the emptiness of the stomach is centrally conveyed and results in an internal state of excitation, of mobilisation of the organism. The newborn child reacts by an undirected motor discharge, in a (vain) attempt to lower the body tension. The baby giggles and screams. Freud (1956/1895a, p. 318) continuous: “The removal of the stimulus is only made possible here by an intervention which for the time being gets rid of the release of *Q*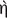 (excitation quantity) in the interior of the body; and this intervention calls for an alteration in the external world (supply of nourishment, proximity of the sexual object) which, as a *specific action*, can only be brought about in definite ways. At first, the human organism is incapable of bringing about the specific action. It takes place by *extraneous help*, when the attention of an experienced person is drawn to the child’s state by discharge along the path of internal state (e.g., by the child’s screaming). When the helpful person has performed the work of the specific action in the external world for the helpless one, the latter is in a position, by means of reflex contrivances, immediately to carry out in the interior of this body the activity necessary for removing the endogenous stimulus. The total event then constitutes an *experience of satisfaction* (… ).” The mother, or another conspecific, hears the cries and thinks “he must be hungry,” she might take the child and put him to her breast. In other words, the mother interprets the cry (or the action) of the child. The child thereby is enabled to release the sucking reflex and milk comes into the body. Thereby, the need is satisfied an this very “removing (of) the endogenous stimulus” is experienced as pleasure according to Freud’s (1955/1920, p. 7–8) definition: “We have decided to relate pleasure and unpleasure to the quantity of excitation that is present in the mind but is not in way “bound,” and to relate them in such a manner that unpleasure corresponds to an increase in the quantity of excitation and pleasure to a diminution.” The milk constitutes an adequate response to the need which was at the source of the discharge impetus (the cry) of the child. Thereby, and thanks to the interpretation, the action acquires a specific status: it becomes an *adequate action* (Freud, 1999/1895b, p. 108).

If the definition of pleasure in this scenario is clear, for Jadin (2012/2009, p. 58), it is also “evident that the endogenous drives (in the experience of satisfaction) constitute an aspect of *jouissance*.” Indeed, Lacan (1986/1959–1960, p. 209) considers that jouissance is the satisfaction of a drive, and not simply of the need which is at its origin. Scherrer (2010, p. 143) even adds: “the aim and object of the drive is pure enjoyment, without an object and unconditionally.” It is at this point that we would like to propose a first operationalisation of the concepts of pleasure and *jouissance*: indeed, we propose that in Freud’s model of the drive ***pleasure is what results from the release of tension induced by the consumption of a suitable object of the drive while jouissance is the (benefit gained from) the motor tension underlying the action which was (once) adequate in bringing relief to the drive.*** In this definition, both, pleasure and *jouissance*, can be aspects of satisfaction of the drive, but, while pleasure implies the consumption of an object, *jouissance* is in the motor mobilization or *use* of the body – i.e., in the motor mobilization of those action pathways that were (once) adequate in delivering pleasure. This definition suits with the juridical origins of the word *jouissance*, where it was reserved for the satisfaction of *using* something without consuming it. Moreover, in the distinction here proposed pleasure is tied to the object, while *jouissance* is related to motor action. This is in agreement with e.g., Marie (2004, p. 27) who says: “*Jouissance* (… ) is very close to l’*Agieren*, (… ) according to its Latin etymology, agere, i.e., accomplish, express by the movement. Any modality of *jouissance* is of the order of the *Agieren*.”

#### Experience of satisfaction

The “experience of satisfaction” is a good place to start discussing *jouissance*, e.g., Marie (2004 p. 25) says: “when the question of enjoyment appears in the writings of Freud, in *The Project*, it is about the experience of satisfaction of the drive economy.” Let’s go back to Freud (1956/1895a, p. 318): “The total event then constitutes an *experience of satisfaction*, which has the most radical results on the development of the individual’s functions. For three things occur in the ψ system: (1) a lasting discharge is effected and so the urgency which had produced unpleasure in ω is brought to an end; (2) a cathexis of one (or several) of the neurons which correspond to the perception of an object occurs in the pallium; and (3) at other points of the pallium information arrives of the discharge of the released reflex movement which follows upon the specific action. A facilitation is then formed between these cathexes and the nuclear neurones.” The *pallium*, in Freud’s vocabulary, is the part of the central nervous system which is connected with the outer body (specific senses and striated muscles) while the *nuclear* neurons innervate the inner body (the viscera)^1^. Freud (1956/1895a, p. 312) adds that “ω is assumed to be filled from ψ ,” in other words, it is the (“nucleus” of the) central nervous system which informs ω^2^ of the actual values of the homeostatic situation in the inner body.

In other words, the experience of satisfaction is as much the adequate resolve of a drive tension than it is the “radical result” of it, namely a lasting facilitation of the associations between a state of body tension at the level of a neural comparator system (ω), a perceptual image of an adequate object, and a motor representation of an action adequate in resolving the tension. As a result: “(… ) when the state of *urgency* or *wishing* re–appears (from ψ to ω), the cathexis will also pass over on to the two memories and will activate them. Probably the mnemic image of the object will be the first to be affected by the *wishful activation.* I do not doubt that in the first instance this wishful activation will produce the same thing as a perception – namely a *hallucination*” (Freud, 1956/1895a, p. 319). We would now add that the wishful activation will also produce a motor body tension, and that this motor tension would then be equivalent to the Lacanian concept of *jouissance*. The biological needs are capable of inducing a reserve of motor tension, which will be recruited to act in order to meet the demands of life, and this reserve of motor tension is equivalent to *jouissance*: “A little bit of *jouissance*, a certain excess is nevertheless necessary from the start. Indeed, the necessities or demands of life (*Not des Lebens*) are such that the nervous system needs to gather a reserve amount to face them” (Jadin, 2012/2009, p. 58).

A question at this point, then, is if this state of body tension is in and by itself in some ways satisfying? It is difficult to decide this question. It could be that body tension has an inherently rewarding effect (see further), but it could also be that the dimension of *enjoyment* more particularly refers to (an) inaugural experience(s) of satisfaction. For example, Scherrer (Freymann et al., 2012, p. 7) says: “The drive is caused by the search, the sting of the recovery of the hallucinatory revival of a previous experience of satisfaction. Hallucination of which we may assume that it was *accompanied by an unprecedented pleasure, particularly intense, excessive, incommensurate with the pleasure associated with the simple release of tension of the need*” (Italics added).

#### Excess of body tension

For Lacan (1986/1959–1960, p. 42; 1965–1966, p. 137; 1999/1972–1973, p. 26), a body “is something that is made to enjoy, to enjoy itself,” “it belongs to a body to enjoy” and “a body is there to be enjoyed.” To understand this, let’s push the Lacanian understanding of *jouissance* a little bit further. In the inaugural experience of satisfaction of hunger, the baby is given the breast by his mother. In a following frontal encounter with the breast, the sucking movement will be released. Jadin (2012/2009, p. 58–59): “But the case may be that the breast is seen from the side. Discharge, then, is delayed and will only take place after a certain search, for example by means of a movement of the head. For this quest, the child must in a first time decompose the perception, this is the *Urteilen* (… ) (the *judging*). The child will perceive at the one hand something identical and specific of the breast, the thing itself of the breast, *the Thing* (*das Ding*) seen from the front, and on the other hand, an element that may vary. When this variable element is strange, the child will delay the discharge. (… ) You can see that *the Thing* is something very specific. It is present at the same time when the object satisfying the drive is effectively perceived, and when the object is only imagined as complete, anticipated by desire. *The Thing* is the portion always invested by the *jouissance* (… ). In the system of neurons described by Freud that *Thing* of the perceptual complex corresponds to a nuclear neuron of the brain which is continuously invested, continuously filled by endogenous quantities, the production of which is constant.”

In our opinion, the notion of jouissance thus seems to balance between two kinds of body tension. The notion of “body tension” is a kind of readiness to act, a motor preparedness, which is probably situated mostly centrally, as an activation level of the central nervous system, but some of which may percolate to the body through subthreshold peripheral motor commands and executions (e.g., mini–contractions). At the one hand, there is the body tension, which we referred to before, specifically preparing an adequate act, which was once accomplished before during an experience of satisfaction. We propose that this part would then correspond to the variable part, as it is activated in reaction to an “attribute” (or an “affordance,” in cognitive term; Gibson, 1977) which functions as a handle for manipulation of the object; as a consequence, it can be represented. At the other hand, there is the body tension, which is induced by the constant “and specific” part of the object, the essence of the thing, *das Ding* itself, which allows it to be identified as such even if the usual attributes for grasping it have changed. What we propose goes as follows: ***as the thing is identified*** – i.e., identified from a past experience of satisfaction, as a potentially satisfying object – ***it induces body tension, which will be needed to act upon it, but*** as the usual “handles” have changed, ***this body tension is yet without clear motor execution form.*** We could say that it has not yet moved (very far) to the motor discharge part of the mental apparatus. Probably this second reading of *jouissance* is closer to Lacan’s (1986/1959–1960) concept of “enjoyment of the Thing.” Jadin (2012/2009, p. 38) says that Lacan presents *jouissance* “in some ways as this which resists to attribution^3^” and further on he adds: “*Jouissance*, the Thing, is thus that which precedes a certain manipulation. It dates from (the time) before the hand” (Jadin, 2012/2009, p. 50). It is (more) easily understood, then, why this jouissance is considered “out–of–representation” (see also Hoffmann, 2012/2009, p. 9): the reserve of body tension has not yet been destined to a determined motor form, which is the basis for representation (see further). Perhaps we could also say it is still very much biological, and not yet mental?

We should also consider the possibility that it is only in this second scenario that, by chance or by surprise, finding the “adequate” way to grasp the object will be “accompanied by an unprecedented pleasure, particularly intense, excessive, incommensurate with the pleasure associated with the simple release of tension of the need,” and that this is what specifically underlies the powerfully satisfying dimension of *jouissance*. But maybe, once found, a new share of body tension shifts to the mental side, where it can be represented. However, the successful mobilization of this share of body tension, though it will be activated at a new encounter with *the Thing*, won’t be able to induce the same extent of gratification as the first time. But at the other hand, if a pathway of discharge is not found, tension may accumulate and this, then, might lead to the experience of pain. Freud (1955/1920, p. 63) says: “Our consciousness communicates to us feelings from within not only of pleasure and unpleasure but also ***of a peculiar tension*** *which in its turn can be either pleasurable or unpleasurable*” (Italics added). Indeed “*jouissance* is suffering” (Lacan, 1986/1959–1960, p. 185) and “What I call *jouissance* – in the sense in which the body experiences itself – ***is always in the nature of tension***, in the nature of a forcing, of a spending, even of an exploit. Unquestionably, there is *jouissance* at the level at which pain begins to appear, and we know that it is only at this level of pain that a whole dimension of the organism, which would otherwise remain veiled, can be experienced” (Lacan, 1966–1967, p. 60; Italics added). We see now also how the pleasure principle thus functions as “a limit to enjoyment” (Lacan, 1991/1969–1970) when it discharges shares of the body tension, bringing relief, and thus pleasure, and limiting the amount of (painful) *jouissance* tension.

### HISTORY

#### Commemoration of a trait, complying to repeat

The radical result of the experience of satisfaction is a facilitation or memory trace “between two mnemic images and the nuclear neurones which are cathected in the state of urgency” (Freud, 1956/1895a, p. 319)^4^. It is the cathexis of the nuclear neurones (which coming from ψ fills ω) which induces a facilitation between the mnemic image of the satisfying object and the (once) adequate motor act. When Freud (1949/1905) says in: “This satisfaction (of a drive) must have been previously experienced in order *to have left behind a need for its repetition*; and we may expect that *Nature will have made safe provisions so that this experience of satisfaction shall not be left to chance” *(Freud, 1949/1905, p. 184; Italics added), we may assume that these “provisions” minimally entail the described inscription of the memory traces. But these are not just passive traces: indeed, they “leave behind a need for their repetition.” Freud (1955/1920, p. 42) explains how this goes in *Beyond the pleasure principle*: “The repressed instinct never ceases to strive for complete satisfaction, which would consist in the repetition of a primary experience of satisfaction (… ) it is the difference in amount between the pleasure of satisfaction which is demanded and that which is actually achieved that provides the driving factor^5^ which will permit of no halting at any position attained, but, in the poet’s words, “ungebändigt immer vorwärts dringt’.^6^” In other words, the traces are not simply sitting there but are continuously activated by the insisting incoming stream from the source of the drive.

Lacan (1991/1969–1970, p. 111–112) comments: “In 1920, what Freud is dealing with in the exploration of the unconscious, is repetition^7^. (… ) Repetition is the denoting, the precise denotation of a trait (… ) being identical to the unary trait, to the little stroke, to the element of writing, of a trait in so far as it commemorates an irruption of enjoyment.” Freud (1949/1905, p. 1212) says, speaking about thumb sucking, that the child is “is determined by a search for some pleasure^8^ which has already been experienced and is now remembered.” Repetition, thus, is the commemoration of an irruption of enjoyment. Lacan (1986/1959–1960, p. 209): “the drive as such is something extremely complex (… ) It embodies a historical dimension whose true significance needs to be appreciated by us. This historical tendency is defined by this, by *this mark, consisting of the drive presenting itself with a certain insistence, in its status of referring* to something memorable because it was remembered^9^. Remembering, “historicizing,” is coextensive with the functioning of the drive in the human psyche.” We can read here the reference to Freud’s facilitation induced by the drive between the two mnemic images, brought about by the experience of satisfaction. This coupling of events forms *a trait or a mark*, commemorating an irruption of enjoyment, and inducing a relentless tendency to repeat: “The compulsion to repeat and (drive) satisfaction which is immediately pleasurable seem to converge here into an intimate partnership” (Freud, 1955/1920, p. 23).

Therefore, taking all this together, we are inclined to think that the experience of satisfaction, having been in itself an experience of *jouissance*, leaves behind a powerful memory trace, which will be readily activated whenever a similar body need or drive situation is aroused, or when “the Thing” is reencountered, thereby inducing a reactivation of the memory images of this (once) satisfying object as well as of the (once) satisfying action. This reactivation will bring about in and by itself *jouissance* through motor tension. This tension might procure (some) enjoyment, especially if some new motor pathway to approach the object has been thought out. Remarkably, this enjoyment then will be released quite independently of the object and action still satisfying the drive from which they historically originated.

## PHYSIOLOGY: THE DOPAMINERGIC PATHWAYS

### BODY

#### Model of the drive: mobilise the external body from within

In the natural history of life, it is with the first vertebrates 520 million years ago that the striated, or *voluntary*, muscles emerge as the system to move the newly invented internal skeleton (see also Bazan, 2008). Vertebrates, then, are schematically constituted of two bodies: an internal body, the invertebrate body, consisting of the so–called vegetative systems for blood circulation, respiration, digestion, excretion, sudation, reproduction etc., and an external body, consisting of the skeleton and the striated or skeletal muscles. These bodies having been in some ways superposed the one upon the other in the course of evolution, for the organism to function efficiently, there must be a system that adjusts the signalling of internal body needs (e.g., oxygen, food, hydration, sex objects) to specific actions of the external body which can alleviate these body needs.

A first physiologic understanding of the Freudian concept of the drive, then, would be the dynamics whereby a body tension, originating from a need in the internal body, mobilises the external body and instigates it to action. One key hypothesis then is that *central dopaminergic systems* could embody the physiological architecture of Freud’s concept of the drive. In the striatum, dopamine (DA) serves as a critical motor action signal; increases in DA are associated with increases in motor output, and decreases in DA with inhibition of behavior. In the case of the mesolimbic pathway, the ventral tegmental area (VTA) innervates the nucleus accumbens shell (NAS), which is part of the corpus striata (basal ganglia); this system is therefore referred to as NAS–DA. This is also the so–called SEEKING* system* of which the neuroscientist Panksepp (1998, p. 145; 144) says that when this system is stimulated: “organisms deploy *the most energized exploratory and search behaviors an animal is capable of exhibiting*: e.g., stimulated rats move about excitedly, sniffing vigorously, pausing at times to investigate various nooks and crannies of their environment,” or else: “The desires and aspirations of the human heart are endless. (… ) But they all come to a standstill if certain brain systems, such as the DA circuits arising from midbrain nuclei are destroyed. (...) These circuits appear to be major contributors to our feelings of engagement and excitement as we seek the material resources needed for bodily survival. (...) Without the synaptic “energy” of DA these potentials remain dormant and still. (...) When DA synapses are active in abundance, a person feels as if he or she can do anything.”

The psychoanalyst and neuroscientific researcher Howard Shevrin has previously made a convincing case that Panksepp’s SEEKING system could stand as a physiological correlate of Freud’s concept of the drive (Shevrin, 2003). Shevrin (2003) indicates how the four parts of the SEEKING system are remarkably similar to the four parts of Freud’s definition of drive and proposes to illustrate this with a simple table (Table 1).

**Table 1:** Shevrin’s (2003) proposition of the parallels between the four parts of Panksepp’s SEEKING system and the four parts of Freud’s definition of the drive.

Panksepp’s SEEKING system	Freud’s drive theory
Regulatory imbalances	Somatic source (*Quelle*)
Consummation	Aim (*Ziel*)
External stimulus	Object (*Objekt*)
Energetic activity	Motor factor (Drang)

Indeed, Panksepp’s SEEKING system is made up of four parts: regulatory imbalances, consummation, external stimulus, and powerful states of expectancy or anticipation, while Freud’s architecture of the drive is also made of four parts. Shevrin (2003) proposes the following parallels: the regulatory imbalances in Panksepp’s model are the underlying specific need states such as hunger, thirst, and sex; thus they correspond with the somatic source of the drive. Consummation refers to the satisfying of the underlying need state, which is what corresponds to the aim of the Freudian drive. External stimulus refers to the object providing the consummatory satisfaction, the most variable component. Concerning the fourth component, Panksepp’s “powerful states of expectancy or anticipation,” they refer to the activation of the NAS–DA. According to Panksepp (1998, p. 145), activation of this system is characterized by a “psychic energization.” When it is activated “… animals perform a large number of motivated goal–seeking behaviors. If this system is damaged, a generalized behavioral inertia results” (Panksepp, 1998, p. 150). Shevrin (2003) indicates that Panksepp’s inference concerning the subjective state of the animal when the NAS–DA circuits is activated, “is based on the intense motor activity of the animal engaging in exploratory activity. In other words the animal is according to Panksepp, energetically active. This clearly implicates a motor factor. Moreover, the activation of the NAS–DA circuits results in an animal engaging in effortful behavior, in Freud’s terms, a demand for work is being made in the most basic meaning of the word work.” In this sense, the “powerful states or expectancy or anticipation” also correspond quite precisely to Freud’s component of motor pressure. We therefore propose that the functioning of the (mesolimbic) dopaminergic pathways could embody a physiological counterpart of Freud’s drive concept^10^.

Let’s again take the case of hunger. Indeed, Panksepp (1998, p. 167) states: “The SEEKING system, under the guidance of various regulatory imbalances, external incentive cues and past learning, helps take thirsty animals to water, cold animals to warmth, hungry animals to food, and sexually aroused animals toward opportunities for orgasmic gratification.” However, if we want to apply this model to the “simple” situation of a hungry baby crying for food the first time, we run into an endless series of complications. First, there are many redundant mechanisms to ensure adequate food consumption any of which may be sufficient to stimulate food intake. Second, the pathways from the internal homeostatic receptor systems detecting various bodily imbalances and inducing the activation of the SEEKING system, i.e., inducing DA–release, are multiple. It is beyond our goal and expertise to give an overview of these, but it seems that the brain architecture underlying appetitive motivation is generally compatible with a drive concept embodied by dopaminergic transmission. For example, if we want to go from hunger to the mobilisation of the external body, the hypothalamus seems a good place to start. It is well established that the arcuate nucleus of the hypothalamus receives humoral signals, both from various nutrients and from various hormones, regarding the status of peripheral energy stores and conveys this information to the lateral hypothalamic area (Elmquist et al., 1999). The LH influences voluntary somatic motor systems governing complex food–searching and food–related behavior. If the LH is activated and food is not present, animals act very aroused, are hyperactive, and appear to engage in searching or foraging behavior (Kelley et al., 2005a,b). Moreover, this LH involvement seems to imply dopaminergic pathways. The lateral hypothalamic corridor between the LH and the VTA is part of the Medial Forebrain Bundle which runs from the VTA to the NAS. The LH also has direct connections with the accumbens shell giving the NAS a privileged access to hypothalamic energy–sensing substrates; however, the LH also more directly reaches widespread areas of striatum (beyond the accumbens) via midline thalamic projection (for review, see Kelley et al., 2005a). In other words, dopaminergic innervation of the striatum, both ventral and dorsal, is involved in food intake, and this system is concerned with motor activation and foraging strategies associated with changing motivational conditions (Haberny et al., 2004; Haberny and Carr, 2005). The complexity of these pathways, however, is huge and there are discrepant opinions in different authors^11^. What is important in the present exercise, is not to show that the dopaminergic transmission is a *necessary* condition for the engagement in appetitive behavior, but to show that the architecture of the brain is broadly *compatible* with the drive mechanism as embodied by dopaminergic transmission, i.e., that it is a possible pathway. Indeed, the first experience of satisfaction, the first cry of the hungry baby may have a quite different physiology as the adult “routine” hunger usually studied in neurosciences. Concretely, when Freud indicates that “The nucleus of ψ is connected with the paths by which endogenous quantities of excitation ascend. (… ) The filling of the nuclear neurones in ψ will have as its result an effort to discharge, an urgency which is released along the motor pathway,” it seems that, given the data summarised above, we are in a position to propose to translate this as: “the central dopaminergic systems are connected with paths which convey information of the internal homeostatic situation of the body, e.g., through the LH. Ascending excitations, indicating, e.g., a food depletion centrally, will lead to release of DA, which will lead to motor mobilisation.”

But how can we now situate the proposed difference between pleasure and *jouissance *in this physiological model? Shevrin (2003) underlines that it has been established across many animal species that once an animal is conditioned to expect a reward following the appearance of a conditioned stimulus such as a light, that at a certain point it will begin to treat the light as if it were the reward itself, in particular if no reward has been forthcoming. A pigeon, for example, will begin to peck at the light even though its pecking has nothing whatsoever to do with the appearance of the reward. This phenomenon is called autoshaping, that is, says Shevrin (2003) “*the animal’s own response, the pecking, becomes intrinsically rewarding.”* When the NAS–DA circuit is artificially blocked with antagonists, the autoshaping disappears (e.g., Di Ciano et al., 2001). Shevrin (2003) comments: “(...) it is not the anticipation of some consummatory pleasure that is involved, a totally different matter, but a pleasure of some sort intrinsic to drive activation. *Consummatory pleasure and, if I may call it that, drive pleasure are two different things*. The first I submit is an emotion or affect in the usual sense; *the second is a unique state of expectation or anticipation that is intrinsically gratifying, but not pleasurable in the usual sense. It is entirely expressed through action, rather than accompanying action* as is the case with consummatory pleasure^12^” [Italics added]. Shevrin’s concept of *consummatory pleasure* seems to parallel Freud’s concept of *pleasure*, resulting from the release of tension induced by the consumption of a “suitable” object (of the drive). Shevrin’s difference between consummatory and drive pleasure therefore parallels quite nicely our own distinction between pleasure and *jouissance*, with *jouissance* defined as the benefit gained from the motor tension underlying the action brought about by the drive, and it allows us to situate the concept of *jouissance* at the level of the intrinsic NAS–DA activation.

These distinctions are also in a remarkable resonance with another neuroscientific distinction. Indeed Berridge (1996), as well as Robinson and Berridge (1993, 2000, 2003), propose a distinction between *wanting* and *liking*. It was first these author’s merit to master two different ways of measuring appreciation in rats: at the one hand, the hedonic (*liking*) or aversive reactions are measured on the basis of facial reactions (some of which are conserved over different species); at the other hand the *wanting* is measured on the basis of the amount of motor activation which the organism is ready to invest in order to obtain the reward. These distinctive parameters allowed for the dissociation of two anatomical circuits (Berridge, 1996): the *wanting* circuit corresponds with the mesolimbic NAS–DA of Panksepp’s SEEKING system, the *liking* circuit corresponds with so–called “opioid hot–spots,” involving among others the shell of the nucleus accumbens, the ventral pallidum and the parabrachial nucleus of the pons in the brain stem. These circuits function independently of each other. For example, considerable research with the taste reactivity test has demonstrated that interference with DA failed to alter appetitive taste reactivity for sucrose (Berridge and Robinson, 1998). Also, enhancing DA neurotransmission is not sufficient to produce pleasurable subjective effects in humans (Rothman and Glowa, 1995). This has led Robinson and Berridge (1993) to conclude that, though the original hypothesis emphasized the role that pleasure played in mediating the effects of dopaminergic manipulations, brain DA does not mediate *liking*. Nevertheless, DA systems are involved in *wanting* of natural and drug reward (see Berridge, 2007); this *wanting* is determined by the intensification of the *wanting* circuits quite independently of *liking*. Indeed, work of these authors shows that activation of DA systems enables or increases behavioral responses necessary for obtaining a goal object, while interference with DA potently affects the willingness of the animal to engage in behavioral actions aimed at anticipating or foraging for food (e.g., Berridge, 1996). It is therefore tempting to draw a parallel between these physiological findings and the psychoanalytic concepts: Berridge’s *wanting* and the psychoanalytic concept of the drive bear some similarities for as far as they both concern the readiness to engage in a motor behavioral effort^13^.

In summary, the proposition that the *central dopaminergic systems*, and in particular the NAS–DA system, could embody a physiological counterpart of the psychoanalytic concept of *jouissance*, seems to be coherent both with the drive–dimension of jouissance and with an understanding of *jouissance* as the benefit gained from the motor tension underlying the action which was (once) adequate in bringing relief to the drive, as Shevrin points out with the phenomenon of e.g., autoshaping. Furthermore, through Shevrin’s distinction between consummatory and drive pleasure, we can see how the Lacanian distinction between pleasure and *jouissance* might reflect or parallel a number of exclusively neuroscience–based distinction, as well as, prominently, the distinction between *liking* and *wanting*.

#### Experience of satisfaction: tag the action associated to a reward

A second aspect of the *jouissance*–NAS–DA convergence would be a convergence around some way of marking the adequate act. The psychoanalytic idea would be that the adequate act, which is also pleasurable, gets is some ways “tagged” during the experience of satisfaction and we have proposed that it is this tagging by experience that will readily reactivate the specific motor pattern when a comparable situation of need is measured by the ω–neurones. The reactivation of this body tension was tentatively understood as *jouissance*. Now, it is well characterised that the presentation of a rewarding stimulus, whose reward value cannot be anticipated, produces a burst of DA firing (Bromberg–Martin et al., 2010). This could fit the psychoanalytic model if some conditions are met. First, the idea of reward should also (partially) cover the Freudian dimension of pleasure, in the sense that the rewarding stimulus should procure tension relief, in particular by being an adequate response to a bodily imbalance situation. Second, it is then this pleasure which should induce the DA release. Third, the effect of this DA should (also) be on the level of the actions involved by stimulus rather than (exclusively) on the stimulus itself and in some ways tag these actions so as to distinguish them from other actions.

First, is the “reward” of the physiological observations comparable to the Freudian pleasure? Salamone et al. (2007, p. 462) define reward as a positive reinforcer with emotional effects, such as feelings of pleasure. The term reinforcer goes back to Thorndike’s (1911) “Law of Effect” which says that “any act which in a given situation produces satisfaction becomes associated with that situation so that when the situation recurs the act is more likely than before to recur also.” This is basically the same as what Freud says for the experience of satisfaction, but Freud gives a criterion for pleasure: he refers to a lasting discharge the information of which is conveyed by the ω neurones, which are filled with the (subcortical and brainstem) neurones in connection with the internal body. So, yes, there is some equivalence between the Freudian concept of “pleasure” and the cognitive concept of “reward” with this proviso that in the cognitive concept no concrete criterion for “satisfaction” is included.

Second, is it then this pleasure which induces the DA release? The neuroscience findings show that it is only when the reward is unexpected that there is a burst of DA firing (Schultz, 1998). So, (Freudian) pleasure does not *per se* lead to DA release, but can it in principle do so, e.g., at some inaugural occasion or in some important need situation? Food in a hungry animal is a very strong reward, but it is probable that here a number of innate criteria (e.g., sweetness) are the triggers for the DA release, and not essentially the relief of the inner body imbalance. However, DA can signal the salience of a variety of potential reward and reward–related cues (Schultz, 1998) and DA appears to play a broad role in motor behavior, rather than a specific role in food intake (Verty et al., 2004; Kelley et al., 2005b). If so, there must be a mechanism beyond the innate which function as a criterion for DA release. It remains unclear how strong, e.g., the role for homeostatic cues of the internal body is in the triggering of DA release, but a contribution of this mechanism is probable given the modulatory role of e.g., satiety on DA release, and should play a more important role for acquired action pathways to find pleasure, or to avoid unpleasure, in complex unanticipable (human) situations.

Now, when encountering such an unexpected reward, DA neurons often produce phasic bursts of activity including multiple spikes (Schultz, 1998). Strikingly, these phasic bursts, which are in this moment perceived as pleasurable (Bromberg–Martin et al., 2010), could be some physiological counterpart of the dimension of *enjoyment* which we have attributed especially to the *inaugural* experience of satisfaction, “*an unprecedented pleasure, particularly intense, excessive, incommensurate with the pleasure associated with the simple release of tension of the need*.” The pleasure here is not to be understood in the Freudian sense, since, indeed, DA and accumbens neurons do not discharge during actual consumption of an expected reward, when the most pleasure is presumably experienced (Schultz, 1992, 1998). It is another kind of pleasure, namely *jouissance*. Strikingly, we are reminded of Freud’s (1955/1920, p. 35) words: “Novelty is always the condition of enjoyment.”

Thus, DA neuron responses are not triggered by reward consumption *per se*, except if the reward was unexpected. Instead, DA neurons discharge *in anticipation of reward* (Koob and Volkow, 2010). Indeed, DA neurons are excited when a cue indicates an increase in future reward value. Alluding to the higher described phenomena of auto–shaping, Volkow et al. (2012, p. 9–10) say that “the mere prediction of a reward may eventually become the reward (… ) this type of functional “switch” has also been reported for natural reinforcers, which are likely to induce an equivalent and gradual shift in DA increases (… ) in the transition from a novel stimulus that is inherently rewarding to that of the associated cues that predict it^14^.” Berridge and Robinson (1998) propose that the phasic DA–bursts *create a state of motivation to seek reward* (see also Salamone et al., 2007). They motivate the individual to obtain the hedonic reward “so that the individual almost cannot sit still” (Berridge, 2007, p. 408). This DA release is necessary for reward cues to cause an increase in general motivation to perform reward–seeking actions (Bromberg–Martin et al., 2010, p. 15). Knutson et al. (2001, p. 271) suggest that the nucleus accumbens “may provide the motivational “engine” that fuels attainment of immediate reward.” This characteristic of behavior has enormous adaptive significance because it enables organisms to exert effort to overcome obstacles or work–related response costs that separate them from biologically relevant stimuli (Van den Bos et al., 2006). We are tempted to make some parallel here between these diverse neuroscience interpretations and Jadin’s (2012/2009, p. 58) psychoanalytic “little bit of *jouissance*” which is nevertheless necessary to face the “demands of life (*Not des Lebens*)” which are such “that the nervous system needs to gather a reserve amount to face them.” In this light, it is interesting to stress that DA systems are activated not only by positive stimuli, but also by aversive, painful and stressful stimuli and events (Berridge and Robinson, 1998; Salamone et al., 2007). Indeed, both rewarding and aversive situations require an increase in general motivation to energize actions and to ensure that they are executed properly. This fits with the clinical observation that *jouissance* can also be tied to actions which were (once) adequate not simply in obtaining pleasure (rewarding situations) but also in avoiding displeasure (aversive situations).

This brings an answer to our third point, namely that the effect of the DA is (also) on the level of the actions involved by the stimulus rather than (exclusively) on the stimulus itself. When Bromberg–Martin et al. (2010, p. 8) say that DA neurons are critical in motivating effort to achieve high–value goals, he adds “and (in) translating knowledge of task demands into reliable *motor performance.*” As a result, the organism will search the stimulus and “*learn actions* to seek it again in the future” (our Italics). Furthermore, Berridge (2007, p. 408) proposes that the DA *tags* the unexpectedly rewarding actions with “incentive value,” which “is a separate form of value added to neural representations of learned signals that predict hedonic reward and which translates the mere prediction into motivation. Incentive salience attribution makes a specific associated stimulus or action into an object of desire^15^ and can tag a specific behavior as the rewarded response the individual is motivated to perform.” Representations of motor processes and cognitive processes are put into chunks in order to mark events as salient and induce appropriate action patterns (Salamone et al., 2007). Although the neural mechanisms of priming are not fully known, “generation of incentive salience is the dynamic process for which mesolimbic DA neurotransmission may be most essential” (Berridge, 2007, p. 412).

Taking all this together, we think we have reasons to see parallels between Freud’s experience of satisfaction and the dopaminergic attribution of incentive salience to reward–related actions. A distinctive feature between the Freudian model and the DA models is that in the Freudian model the instigation (for motor activation) is more readily understood as coming from within the organism, originating, e.g., from some homeostatic imbalance situation, pushing to go find reward, while in the DA models the instigation is induced by some perceived stimulus, potentially announcing a reward. However, one could conceive of both models as three–way connexions both implying all three, the bodily need, the perceived stimulus or object^16^, and the motor pathway to grasp it or interact with it. Indeed, Berridge (2007, p. 414, 413) states it as follows: “the mesocorticolimbic circuitry (… ) mediates the integration of learned signals with hunger/satiety states to dynamically transform the motivational value of stimuli” or even more directly: “physiological deprivation states (… ) motivate and direct (behavior) chiefly by enhancing the motivational and hedonic values of their relevant external incentive stimuli and that is a function for which mesolimbic mechanisms may be important.”

This last sentence resonates with “when the state of urgency or wishing re–appears, the cathexis will also pass over on to the two memories and will activate them,” the two memories being the rewarding object or incentive stimulus and the motor pattern of its associated behavior. As indicated, the precise role of DA release in this dynamic is to fuel the organism by creating a state of motivation to seek reward, and this again is strikingly close to the definition of *jouissance* we have proposed in the framework of the experience of satisfaction, namely the motor body tension instigated by the wishful activation. We can also hear quite directly the neuroscience connexion between reward and motivation in Freud’s (1955/1920 p. 23) statement: “The compulsion to repeat and (drive) satisfaction which is immediately pleasurable seem to converge here into an intimate partnership.”

#### Excess of body tension: induce excess to the point of exhaustion

If *jouissance* is to be understood as equivalent to a state of motor activation, then, by consequence, it is also equivalent to an increase in body tension. Indeed, any action intention, be this action actually executed, or simply imagined, remembered, prepared, anticipated or even prevented (e.g., Jeannerod and Decety, 1995; Decety, 1996; Gallese, 2000), leads to a slight increase in muscle tension (ex. Yue and Cole, 1992). The actions which are actually executed are only a fragment of all motor activations continuously mobilising the body and causing muscle tension. The idea of *jouissance* as (the benefit from) a state of motor activation, therefore leads us directly, following a physiological logic, to the idea of sustained high levels of body tension, corresponding to what seems to be put forward by Lacan as central in the concept of *jouissance*, namely the implication of the body.

What then about Lacan’s proposition of the closeness between *jouissance* and the notions of excess and pain? It is interesting, in this respect, to remember the first observations implying the stimulation of the nucleus accumbens. Indeed, in a well–known series of experiments, Olds and Milner (1954) devised a system enabling a rat to stimulate its own brain, by means of a lever connected to an electrode implanted in the forebrain. Olds and Milner (1954) describe that rats would continually press the lever in return for receiving nothing more than a brief pulse of electrical stimulation. It turned out that a similar effect was also produced when the electrodes were implanted in the nearby nucleus accumbens (Olds, 1956). The rats would press the lever frequently to receive stimulation and would work so vigorously to self–administer stimulations to the point of exhaustion and the exclusion of all other activities (e.g., eating drinking, sex, and sleep). When, in the wake of certain surgical procedures, similar stimulations were possible for some human patients, it was indeed also observed that these patients preferred this self–stimulation above all other activities. But, curiously, this stimulation was not associated with any external sign of pleasure: no smile or relaxed face, or any other sign of tangible happiness, or subjective expression of a pleasant sensation (Berridge and Kringelbach, 2008, p. 15). It is for this reason then that the term “reward” circuit was chosen rather than Olds and Milner’s (1954) first description of “pleasure center.” What is moreover striking is that is again the same neurophysiologic axis, the NAS–DA, which is implied here and that it reveals itself as an axis which could, if circumvented (or “perverted;” ex. by self–stimulation), easily lead to excess to the point of exhaustion and self–harm.

To further strengthen this idea that the same mechanism, so vitally important in driving the organism and in tagging adequate actions, is also the mechanism which easily shifts toward harmful effects, let’s go back to the role of the NAS–DA in inducing body tension. Indeed, the situation of self–stimulation is artificial and therefore not common. However, let’s remember that DA is not generally released during the consummatory phase, but in advance of it, inducing a state of motor tension leading the organism to move toward the rewarding stimulus. In that sense it is interesting to note that, e.g., Kupfermann et al. (2000) comment that this anticipation, or motor tension, translated by the mesolimbic DA firing, can be interpreted as a deficit, inducing *an anxiogenic state of tension*, rather than being already rewarding *per se*. For all these reasons, we contend to say that in the same way as for *jouissance* where the boundary between enjoyment and pain seems flimsy, the mesolimbic NAS–DA functioning might be so built that it is in a constant instable balancing between reward and anxiety. Moreover, in both cases it is the part of the body tension which actually can go into effectively executed action, and therefore into discharge, which limits the build–up of tension, and therefore of pain or anxiety. This then could be some physiological counterpart of the psychoanalytic idea of pleasure functioning as a limit to *jouissance *(Lacan, 1991/1969–1970).

### HISTORY

#### Commemoration of a trait, complying to repeat: incentive sensitization

As concerns the history dimension of *jouissance*, we will elaborate on Robinson and Berridge’s “incentive sensitization” theory since this theory not only entails the tagging effect of DA release but more specifically the *structural* inscription aspects of the DA reward system and therefore seems to be in an ideal position to translate the specifically *historical *dimension of *jouissance. *Indeed, when Robinson and Berridge first present their incentive sensitization theory of addiction in 1993, they proposed that the most important of the psychological changes in addiction is a “sensitization” or hypersensitivity, i.e*., long–lasting adaptations* in the mesolimbic NAS–DA. Addictive drugs share the ability to produce persistent neuroadaptations that render these regions hypersensitive. The data suggest that sensitization may involve more than a simple up– or down–regulation of biochemical processes, but it may involve changes in patterns of synaptic connectivity in brain reward systems, changes that may be similar to those seen in other neural systems in association with other forms of experience dependent plasticity (Robinson and Kolb, 1997, 1999). This is accompanied by an increase in spine density on the distal dendrites of these cells. These neuroadaptations in DA/accumbens systems specifically, then produce a pathological motivation for drugs, called compulsive “*wanting.*”

Several points of this incentive sensitization theory are important in the current perspective. First, as a consequence of the dissociation between *liking* and *wanting*, the authors stress the fact that this theory does not simply account for the addiction by the positive and/or negative reinforcement value of the drugs, i.e., the addiction is not simply due to the desire to experience the positive hedonic effects of the drugs and/or to avoid aversive withdrawal symptoms, as proposed in other theories (e.g., Koob et al., 1989; Markou et al., 1993). The incentive salience theory explicitly shifts the hypothesis away from the conjonctural reinforcement aspect toward the structural alterations aspects. For example, Robinson and Berridge (2000, p. S96) state: “Perhaps the most remarkable feature of sensitization is its persistence. Once they have been sensitized, animals may remain hypersensitive to the psychomotor activating effects of drugs for months or years.” In other words still, it is clear that the *wanting* circuit not only operates as a driving, and sometimes rewarding, system, but it is also sensitive to long lasting adaptations, i.e., to *historical imprint*. This, then, is coherent with the historical dimension of *jouissance* defined by “this mark, consisting of the drive presenting itself with a certain insistence, in its status of referring to something memorable because it was remembered” (Lacan, 1986/1959–1960, p. 209).

Further, the incentive sensitization theory also includes several aspects, which makes this theory a truly *psychological* theory. First, the theory of Robinson and Berridge (2000, p. S105) fully acknowledges the functional status of *representations* in this incentive salience process, e.g., “It is further hypothesized that the psychological process that leads to “*wanting*” involves the attribution of attractive salience to stimuli *and their representations*, a process we call incentive salience attribution. (...) We have suggested it is the process of incentive salience attribution that transforms the sensory features of ordinary stimuli or, more accurately, *the neural and psychological representations* of stimuli, so that they become especially salient stimuli, stimuli that “grab the attention,” that become especially attractive and wanted, thus approach and guiding behavior to the goal.” This role for representations is also logical in a action–centred rather than a stimulus–centred perspective, since it is known that preparation of an action as well as anticipation, imagination, remembering etc. of that action share a common motor imagery (Decety, 1996) and that this imagery could be the substrate of its representation (Jeannerod, 1994). Second, these representations can also be *unconscious*. For example, in addicts, doses of drugs that are too low to produce any conscious experience of pleasure can activate implicit *wanting* as indicated by an increase in drug–seeking behavior. Robinson and Berridge (2000, p. S104) propose: “the incentive–sensitization theory holds that drugs can activate positive core processes of motivation in the absence of conscious awareness, so that positive effects may not be indicated on any scale of subjective affective intensity. Indeed, the neural system responsible for incentive salience attribution can sometimes produce *wanting*, in the absence of conscious awareness of *wanting* itself (Robinson and Berridge, 2000, p. S105; see also Berridge, 1996, 1999). For example, the brief subliminal (i.e., unconscious) presentation of faces expressing positive emotions can activate implicit *wanting* increasing subsequent consumption of a beverage (Berridge and Winkielman, 2003). Robinson and Berridge (2000, p. S106) add: “Activation of this system (...) can act sometimes as an unconscious motivational process.” In other words, the incentive salience theory can account for *an unconscious representation unconsciously inducing an intentional body investment or motor tension* – which is, in our opinion, also a highly psychoanalytic idea*.* The sensitized pathways, the neuroadaptations in the *wanting* system, are not simply sitting there, but form an active past, which has the continuous potential to press for action., i.e., which “unsubdued, pushes ever forward.”

In this incentive salience framework, the clinical link between *wanting* and *jouissance* is quite direct: when the *wanting* system is activated implicitly, it can instigate and guide behavior without a person necessarily having conscious emotion, desire, or a declarative goal (Robinson and Berridge, 2003, p. 36). This kind of perplexity pertains clinically to a whole variety of behaviors which people persist in having, even if they are neither pleasurable, reasonable nor desirable, and even if they are negative or destructive. This is keenly observed in clinics: addicts maintain their consumption while they “may report they are miserable, their life is in ruins, and that even the drug is not that great anymore. They are themselves bewildered by the intensity of their own compulsive behavior^17^” (Robinson and Berridge, 2000, p. S106). Often the addict describes his behavior as simply an overwhelmingly strong craving that cannot be denied. Strikingly, these are the very type of clinical observations which have originally led to the necessity of thinking the concept of *jouissance* in psychoanalysis. Moreover, addiction has often been the terrain *par excellence* for the psychoanalytic description of *jouissance* (Braunstein, 1992; Delourmel, 2009), with as a basic tenet that it is not the pleasure of the consumption but the *jouissance*, which ties the subject to his addiction. Robinson and Berridge (2000, p. S105) understand reward starting as the combination of *liking* and *wanting* but stress that unlike the wanting systems, the neural systems that mediates the subjective pleasurable effects of drugs do not appear to sensitize. They add: “This may be why addiction is characterized by an****increasing dissociation between the incentive value of drugs (how much they are wanted) and their subjective pleasurable effects (how much they are liked). With the development of an addiction drugs become pathologically wanted (“craved”) and this can occur even if drugs are liked less and less.” Again, the parallel with a psychoanalytic reading is striking: the reward as the combination of *liking* and *wanting* could correspond to the inaugural moment of *jouissance*, marking the adequate act which also brings pleasure. The dissociation between pleasure and enjoyment, moreover, could resonate with so–called *morbid jouissance* which accounts for the persistence of behaviors, which may^18^ once have been adequate. However, even if they are no longer pleasurable, since they were effectively registered in memory, they will not fade away but will persist and push for their repetition, while most often remaining beyond conscious understanding (see also Johnson, 2008). Indeed, as said (see Commemoration of a Trait, Complying to Repeat), *jouissance* can persist independently of the object and action still being adequate in respect to the body need or drive from which they historically originated.

## CONCLUSION

In other words, it seems that the parallels between the different dimensions of the psychoanalytic concept of *jouissance* and the different aspects of the NAS–DA physiology are quite striking. At the level of the body, the NAS–DA has been proposed to function as ^18^ a basic *drive* system much in the same way as described by Freud; *jouissance* then arises when this system goes awry, namely when the action is invested in and for itself, which is structurally bound to happen, as is shown in the phenomena of e.g., autoshaping. The NAS–DA is also the body system which tags actions which have brought (unexpected) reward and as a result of this tagging, a new encounter with the incentive stimulus will fuel a reserve of body energy motivating the organism to search the reward (or to avoid the aversive situation). This highly resonates with Freud’s experience of satisfaction where either a bodily need, or a new encounter with “the Thing,” will reactivate the mnemic motor image for action upon this “Thing.” The tension induced by this reactivation, again, we have referred to as *jouissance*. Third, the mesolimbic NAS–DA is also the axis which functions in a constant instable balancing between reward and anxiety, reflecting the flimsy boundary between enjoyment and pain described for *jouissance*. At the level of the (organism’s) history, the NAS–DA is the central operator in the so–called incentive salience theory, which describes how neuroadaptations due to reward can sensitize selectively the *wanting* system while leaving the hedonics or *liking* unchanged. This theory could therefore account for Lacan’s historical dimension of *jouissance* defined as a mark referring to something memorable and commemorating an irruption of enjoyment. In both theories the memory traces relentlessly push for action, i.e., push for their repetition, which can explain the perplexifying persistence of behavior while it is no longer pleasurable, and even when it becomes damaging, such as in addiction. For all these reasons, *jouissance* could be described as an accumulation of body tension, fuelling for action, but continuously balancing between reward and anxiety, and both marking the physiology of the body with the history of its commemoration and arising from this inscription as a constant push to act and to repeat. Moroever, it seems that the mesolimbic NAS–DA is a reasonable candidate for its underlying physiological architecture.

## Conflict of Interest Statement

The authors declare that the research was conducted in the absence of any commercial or financial relationships that could be construed as a potential conflict of interest.

## Footnotes

1 Note that this roughly corresponds to the divide between the brainstem and limbic system, innervating the vegetative functions of the internal body and the neocortex, innervating the sensory systems and the motor control of the external body.

2 Freud’s ω–neurones are a real headbreaker: they are “excited along with perception” Freud (1956/1895a, p. 309), “behave like organs of perception, and in them we could find no place for a memory” Freud (1956/1895a, p. 309) while “These neurones must have a discharge, however small (… ). The discharge will, like all others, go in the direction of motility (… ).” Freud (1956/1895a, p. 311). Freud (1956/1895a, p. 309) *needs* to think them to understand how physical entities (e.g., stimuli), which structurally are *quantities*, become mental entities characterized by *quality*. As to their working principle, Freud proposed that they are incapable of receiving stimuli, “but that instead they appropriate the *period* of excitation and that this state of theirs of being affected by period while they are filled with the minimum of *Q* [quantities, stimuli] is the fundamental basis of consciousness” Freud (1956/1895a, p. 311) and indeed “consciousness is the subjective side of one part of the physical processes in the nervous system, namely of the ω processes.” We propose that these ω–neurones could stand for *comparator systems* in general, which very much work like thermostats comparing set–values with actual values (see also Bazan, 2007). They allow absolute quantity amount to acquire, by comparison, value labels (e.g., “so much short of,” “so much in excess of,” etc.), i.e., *they transform quantities in qualities*. The confusion about them being sensory or motor would be logical as they comprise a sensory monitoring directly connected to a possible (disinhibition of) discharge. Moreover, Freud adds: “*unpleasure* would have to be regarded as coinciding with a raising of the level of *Q* or an increasing quantitative pressure; it would be the ω sensation when there is an increase of *Q* in ψ . Pleasure would be the sensation of discharge.” We are tempted to translate this as follows: at the level of the comparator the more the set value and the actual value differ, the more this is unpleasurable, while when they coincide, there is discharge and, consequently, relief, which is pleasurable. And then: “Pleasure and unpleasure would be the sensations in ω of its own cathexis, of its own level; and here ω and ψ would, as it were, represent intercommunicating vessels. In this manner the quantitative processes in ψ too would reach consciousness, once more as qualities” (Freud, 1956/1895a, p. 312). The idea of “intercommunicating vessels” could fit with these comparator systems which are, indeed, continuously balancing neurons firing for target values (e.g., efference copies) and afferent information which has already gone through ψ , the central nervous system. The cryptic “period” which Freud refers to, could tentatively be understood as the intensity of firing of the one and the other type of neurons, i.e., at the level of the neural comparator systems the only point which still matters is to balance out the intensity levels of both informations.

3 “Referring to the two judgments which must be applied toward a thing, the judgment of existence and the judgment of attribution, Freud holds that one can assign an infinite number of attributes to a thing, to *The Thing*, to the *ousia*. When Lacan argues that *jouissance* is *ousia*, he presents it in some ways as this which resists to attribution, as that which is excluded from the attribution judgment (… ).”

4 Freud explains a mechanism of how such a memory trace may come about: “Now there is a basic law of *association by simultaneity*, which operates in the case of pure ψ activity, of reproductive remembering, and which is the foundation of all links between the ψ neurones. We find that (… ) *quantitative cathexis of a ψ neurone, α , passes over to another, β , if α and β have at some time been simultaneously cathected* from Φ (or from elsewhere) [Italics added]. Thus *a contact–barrier has been facilitated through the simultaneous cathexis α –β *.” – with Φ, being the “permeable” neurones at the periphery of the mental apparatus, both perceptual or motor neurones. Note that Freud’s description is equivalent to Hebb’s (1949) law and explains how the easy connexion between two neurones comes about by being first both simultaneously activated from elsewhere.

5 Freud seems to allude to the idea that this incoming stream is compared with a set value in a comparator type monitoring mechanism such as what we have proposed for ω.

6 “Presses ever forward unsubdued.” Mephistopheles in *Faust*, Part 1 [Scene 4].

7 Our translation of “En 1920, ce à quoi Freud a affaire dans l’exploration de l’inconscient, c’est la répétition.” Which we prefer above the official translation which is “This is even what Freud discovered precisely around 1920.”

8 This pleasure here is *jouissance*, the enjoyment of the body tension which was once adequate in bringing pleasure.

9 Our translation of “Cette tendance historique se définit en ceci, dans cette marque que la pulsion se présente dans une certaine insistance, en tant qu’elle se rapporte à quelque chose de mémorable parce que mémorisé.” which we prefer above the official translation which is “This dimension is to be noted in the insistence that characterizes its appearances; it refers back to something memorable because it was remembered.”

10 Other neuroscientific authors have also made propositions in the same line, though probably not with *Freud*’s concept of the drive in mind: e.g., Kupfermann et al. (2000, p. 998) in their seminal work Principles of neural science propose that the mesolimbic DA transmission appears to represent a state of motivation or drive wherein drive states can be understood as a state of tension due to a physiological need or homeostatic imbalance. Drives direct behavior toward a goal, able to reinstall homeostasis via consumption of an object (e.g., food).Moreover, they increase general alertness, energizing an individual to act appropriately in a given situation, in order to obtain a goal.

11 For example, even if a specific role for the medial accumbens shell in food-directed behavior and food consumption was indeed demonstrated (Stratford and Kelley, 1997; Reynolds and Berridge, 2001), Kelley etal. (2005a) sums up a number of results which seem difficult to reconcile with the idea that dopaminergic transmission is necessary for appetitive approach.

12 Interestingly, Salamone et al. (2007, p. 465) summarize in a footnote a number of comparable distinctions which several authors have made between two kinds of gratifications: e.g., activational versus directional; preparatory versus consummatory; instrumental versus consummatory; anticipatory versus consummatory; ethanol seeking versus ethanol intake; anticipatory versus hedonic.

13 Similarly, Robinson and Berridge’s *liking* and the psychoanalytic concept of *pleasure* bear some similarities for as far as they both concern a pleasure or hedonic experience upon consummation of an object. However, *pleasure* in the Freudian sense is defined as a relief of body tension due to the satisfaction of a bodily need. *Liking*, at the other hand, is brought about by stimulation of the opioid hot–spots which react by innate pathways to the “orosensory properties” (i.e., its sweet taste and fatty flavors), to some extent independently from the information on the homeostatic situation of the internal body (Sclafani, 2004). Of course, this schema works for as long as we stick with “basic” drives which are so fundamental to life and conserved over evolution that they have strong innate foundations. But there are many other internal body tensions which can arise in the course of our first interactions with our primary caregivers besides hunger, especially in the human species – which of all species is the most dependent of his first caregivers. It would be interesting to find out if homeostatic relief, in general, can lead to opioid activation and if this opioid activation could serve as a criterion for the dopaminergic tagging of the associated action. Different elements point in that direction, since, e.g., the homeostatic state can modulate the rewarding value of a stimulus, but the total picture remains unclear. Now, for the model to function beyond more innate drives like hunger (where associated criteria like sweetness may suffice as a criterion for the adequacy of action), i.e., for the model to function in more typically human situations, the dopaminergic reward system should be able to take into account the situation of the internal body as a criterion for tagging actions. In this sense the NAS–DA being informed of this homeostatic information is sufficient (see further) and the opioid activation may or may not constitute a correlate of the Freudian concept of pleasure.

14 This transition is conveyed through DA signalling, which appears to code for a so-called “reward prediction error,” which has been proposed to act as a teaching signal that underlies reinforcement learning (Schultz, 1998). However, Berridge (2007), on the basis of a series of empirical observations, refutes the causal role of DA in (reinforcement) learning and proposes that, in order to explain why mesolimbic dopamine neurons so elegantly fire seemingly obeying prediction error equations, they code an informational *consequence* of learning signals, *reflecting* learning and prediction generated elsewhere in the brain rather than causing it. Salamone etal. (2007) also distinguish a possible role for DA in learning from its motivational or reward role.

15 We can’t help but point out that even Berridge’s word choices become properly psychoanalytic at times.

16 which would be “the Thing” in the psychoanalytic reading, see Excess of Body Tension: “as the thing is identified – i.e., identified from a past experience of satisfaction, as a potentially satisfying object – it induces body tension, which will be needed to act upon it.”

17 And the authors add this very psychoanalytic observation: “Indeed, addicts probably have no more insight into what motivates their daily behavior than do the rest of us; which is arguably, not much.”

18 *Or may not*: Indeed, in *Beyond the pleasure principle* Freud (1955/1920) explains how a subject may also comply to repeat actions which were never pleasurable or adequate in the first place. He further indicates that the binding effect, which is a first necessary step toward discharge, is in this repetition probably the decisive element. In other words, acting upon a traumatic experience, even if this action is inadequate in bringing relief, is, in and by itself better than sideration, because the mere action channels the threatening accumulation of quantities toward a dischargeable motor form.
